# Caspofungin therapy in prosthetic valve endocarditis and candidemia due to itraconazole-resistant *Candida glabrata* (*Nakaseomyces glabratus*): A case report

**DOI:** 10.22034/cmm.2025.345248.1615

**Published:** 2025-08-03

**Authors:** Mohamad Rafi Khurgami, Mahsa Fattahi, Effat Hosseinali Beigi, Pegah Tamimi, Aliasghar Ghaderi, Golnaz Hajiesmail

**Affiliations:** 1 Rajaie Cardiovascular Medical and Research Center, Iran University of Medical Sciences, Tehran, Iran; 2 Immunology, Asthma and Allergy Research Institute, Tehran University of Medical Sciences, Tehran, Iran; 3 Children's Medical Center, Pediatrics Center of Excellence, Tehran University of Medical Sciences, Tehran, Iran; 4 Division of Pediatric Intensive Care Unit, Bahrami Children Hospital, Tehran University of Medical Sciences, Tehran, Iran; 5 School of Medicine, Tehran University of Medical Sciences, Tehran, Iran

**Keywords:** Azole resistant, Blood-stream infection, Endocarditis, *Candida glabrata*, *Nakaseomyces glabratus*, Caspofungin

## Abstract

**Background and Purpose:**

Candidemia is a prevalent nosocomial bloodstream infection with a high mortality rate. Involvement of heart valves by *Candida* spp. after candidemia can result in native and prosthetic valve endocarditis as biofilm-related infections.

**Case Report:**

This report aimed to introduce a case of a 13-year-old male with bloodstream infection and prosthetic valve endocarditis, caused by itraconazole-resistant *Candida glabrata* (*Nakaseomyces glabratus*). Despite undergoing itraconazole for 4 weeks, the patient did not improve.

White colonies were identified as *C. glabrata* (*N. glabratus*) by Restriction Fragment Length Polymorphism-Polymerase Chain Reaction. The isolate was resistant to itraconazole (MIC=8 µg/mL) but susceptible to amphotericin B and caspofungin. Based on concerns about biofilm-related resistance, treatment was switched to caspofungin for 5 weeks. He continued to do well and showed no signs of relapse during his 6-month follow-up.

**Conclusion:**

This study explored the importance of antifungal susceptibility testing in handling complicated infections, as well as the potential of caspofungin in the treatment of cases of fungal bloodstream infections and endocarditis.

## Introduction

Candidemia is a prevalent nosocomial bloodstream infection with a high mortality rate (up to 40%) [ 1 - 3 ]. Fungal endocarditis or colonization of heart valves by *Candida* spp. after candidemia can result in both native valve and prosthetic valve endocarditis as biofilm-related infections [ 4 ].

*Candida albicans* is the predominant species, but non-*albicans Candida* spp. (NAC spp.) are increasingly reported, posing challenges due to their unfamiliar pathogenicity and limited diagnostic options [ 5 , 6 ]. The rise is linked to a growing population of vulnerable patients, including immunocompromised individuals and those undergoing major surgeries [ 7 , 8 ] as well as excessive use of broad-spectrum antifungals [ 9 ]. *Candida glabrata*, which is also referred to as *Nakaseomyces glabratus*, is recognized for causing severe fungemia and has the ability to disseminate throughout the body, particularly targeting heart valves [ 10 ].

Accurate species identification is essential for targeted antifungal therapy [ 11 ]. Blood culture identification is widely recognized as the gold standard approach in medical laboratories [ 12 ], but these methods are time-consuming, delaying effective management. Therefore, there is a need for faster molecular techniques, such as Restriction Fragment Length Polymorphism-Polymerase Chain Reaction (RFLP-PCR).

## Case Report

This case report was conducted in 2022 at Rajaie Cardiovascular Medical and Research Center, Iran University of Medical Sciences, Tehran, Iran, and the Immunology, Asthma, and Allergy Research Institute, Tehran, Iran. All practices followed the Helsinki Declaration; accordingly, informed consent for both treatment protocol and publication was obtained from the patients and parents. The Ethics Committee of Shahid Rajaee Hospitals, Tehran, Iran, approved this study (IR.TUMS.SPH.REC.97000).

A 13-year-old Persian boy with a history of prosthetic valve replacement six weeks ago was referred to our center. The patient underwent prosthetic valve replacement due to congenital bicuspid aortic valve stenosis with severe left ventricular outflow obstruction, diagnosed during cardiologic evaluation for exertional dyspnea and chest discomfort. A mechanical valve was selected considering the age of the patient (13 years) and the need for long-term durability, in accordance with pediatric cardiothoracic surgery recommendations. Postoperative recovery was initially uneventful.

His symptoms, including fever of unknown origin and fatigue, began four weeks ago. At initial presentation, his vital signs were as follows: temperature: 38.9 °C, heart rate: 118 bpm (tachycardia), respiratory rate: 22 breaths/min, blood pressure: 95/60 mmHg, and oxygen saturation: 96% on room air. While hospitalized, his inflammatory markers confirmed creatinine at 0.4 mg/dl (NR: 0.7-1.6), SGOT at 75H IU/L (NR 7-38), and SGPT at 85H IU/L (NR 7-38). The presence of *Candida* spp. was confirmed in three sets of blood cultures, with transthoracic ultrasound (TTE) showing prosthetic valve malfunction. The TTE demonstrated abnormal motion of the prosthetic aortic valve leaflets, suggestive of prosthesis dysfunction. The TTE also identified irregular, mobile echodensities attached to the valve, with sizes up to 8 mm, compatible with fungal vegetations. There was also evidence of moderate paravalvular leak and increased transvalvular gradient, consistent with valve dehiscence or dysfunction, which raised concern for prosthetic valve endocarditis.

Identification of fungal vegetations prompted the immediate start of empiric therapy with itraconazole at a dose of 150 mg/kg/d. Initially, itraconazole was selected empirically due to the limited availability of liposomal amphotericin B and echinocandins in our center at the time of diagnosis. Additionally, concerns about nephrotoxicity in a pediatric patient with borderline liver enzyme abnormalities influenced the choice. Fluconazole was avoided due to possible *C. glabrata* (*N. glabratus*) or *C. krusei* involvement. Once susceptibility testing confirmed resistance to itraconazole and the identification of *C. glabrata* (*N.glabratus*), the antifungal regimen was promptly switched to caspofungin, which was later made available through hospital procurement. Positive blood cultures were detected on the seventh, 21^st^, and 30^th^ day of antifungal treatment. Following four weeks of treatment with standard dose and duration of itraconazole, the clinician requested mycological testing (including *Candida* spp. identification and antifungal susceptibility testing) due to the lack of clinical progress.

### Mycologic Examination

Blood samples were cultured in biphasic medium (Thermo Fisher, USA) and incubated at 37 °C. Isolates were subcultured on CHROM agar *Candida* medium and incubated for 24 h. The DNA was extracted using the Roch DNA Extraction Kit (Germany), and RFLP-PCR was performed with ITS1-F and ITS4-R primers (The ITS1-4 primers ITS1-F [5’-TCC GTA GGT GAACCT GCG G-3’] and ITS4-R [5’-TCC TCC GCT TAT TGA TAT GC-3’] were employed in PCR amplification) using an Eppendorf thermal cycler . Amplicons (400–900 bp) were analyzed on 1.5% agarose gels stained with DNA-safe dye.

To achieve accurate discrimination of *Candida* spp., the amplified product was treated with the specific restriction enzyme MspI. Colorless colonies were detected on chromogenic media, and the fungal pathogen was recognized through its electrophoretic pattern. Antifungal Susceptibility Testing (AST) was performed on planktonic cells using Clinical and Laboratory Standards Institute M27/M60 broth microdilution [ 13 ], testing itraconazole, amphotericin B, and caspofungin (Sigma-Aldrich, USA). *Candida parapsilosis* ATCC 22019 served as quality control.

*Candida* biofilms were grown on 96-well microplates, and AST was performed on planktonic cells as described earlier. After incubation, planktonic cells were removed, and biofilms attached to the wells were washed once with sterile phosphate-buffered saline. Antifungal agents were applied in 100 µl doses and incubated for 24 h at 37 °C. Biofilms were then treated with 100 µl of 3-[4,5-dimethylthiazol-2-yl]-2,5 2,5-diphenyl tetrazolium bromide solution for 3 h at 37 °C. Experiments were repeated at least twice or three times. Test components included 1X phosphate buffered saline (negative control) and antifungal concentrations ranging from 0.03% to 3.2%.

**Figure 1:**
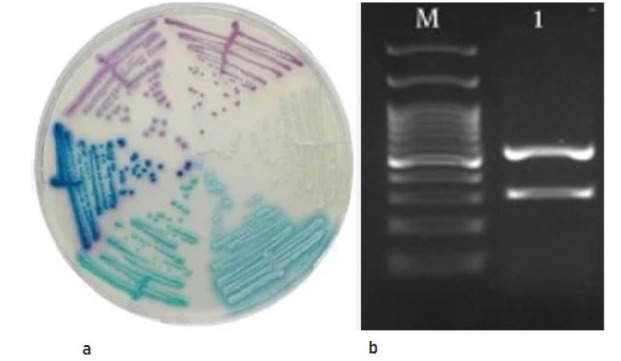
**a.** White colony of *Candida* spp. on CHROM agar *Candida* media; **b.** Restriction fragment length polymorphism pattern of a white colony of *Candida glabrata* (*Nakaseomyces glabratus*); M: DNA marker

Following incubation on CHROM agar *Candida* media, white colonies suggested *C. glabrata* (*N. glabratus*) or closely related species, confirmed by RFLP-PCR with two fragments (557 bp, 314 bp) indicative of *C. glabrata* (*N. glabratus*) (Figure 1).

Table 1 presents the minimal inhibitory concentrations (MICs). The white colony isolate of *C. glabrata* (*N. glabratus*) showed susceptibility to both amphotericin B and caspofungin. Planktonic cells that were unresponsive to itraconazole treatment in clinical antifungal therapy were classified as resistant to itraconazole based on the MIC of the strain tested (MIC= 8 µg/mL).

**Table 1:** Antifungal susceptibility pattern of *Candida glabrata* (*Nakaseomyces glabratus*) isolate with white colony color on CHROMagar *Candida* media.

Isolates	MIC(µg/mL)			
	Itraconazole	Voriconazole	Amphotericin B	Caspofungin
*Candida glabrata* (*Nakaseomyces glabratus*)	8	0.25	0.125	0.625

MIC: Minimum inhibitory concentrations

Considering that *C. glabrata* (*N. glabratus*) can adhere to tissues or a prostatic device and create a complex biofilm structure, the likelihood of biofilm formation should be acknowledged as a significant factor in treatment resistance. The colony-forming unit did not show a significant decrease following itraconazole treatment. Conversely, treatment with amphotericin B and caspofungin caused a notable drop in the colony-forming unit quantity. The collected data implied that resistance could develop during treatment as a result of biofilm formation. Based on the antifungal susceptibility findings, the treatment was switched to caspofungin (3 mg/kg/day) for a duration of 5 weeks.

## Discussion

Through the implementation of the AST and biofilm antifungal susceptibility assay, we effectively treated bloodstream infection and endocarditis. The patient showed poor clinical and in vitro response to itraconazole, and biofilm cells exhibited azole resistance. This is among the few pediatric cases of prosthetic valve endocarditis by *C. glabrata* (*N. glabratus*), a rare pathogen in this setting. This is one of the few documented pediatric cases of prosthetic valve endocarditis caused by *C. glabrata* (*N. glabratus*), which itself is a rare pathogen in this setting and age group.

Novelty of this report lies in the use of a dual approach combining molecular identification (RFLP-PCR) and antifungal susceptibility testing on both planktonic and biofilm forms, providing a comprehensive understanding of treatment resistance. Considering biofilm susceptibility may help clinicians adjust therapy when standard treatment fails.

As a result, the use of antifungal susceptibility testing on both planktonic and biofilm forms is a valuable approach for individuals infected with *Candida* spp. [ 14 ] to promptly identify the suitable medication.

Findings of this study emphasized that resistance to itraconazole may be driven by biofilm formation, which is often underdiagnosed in standard clinical evaluations. This approach aims to minimize the unnecessary exposure of susceptible yeast to medications, thereby stopping additional changes that lead to drug resistance. [ 15 ]. It is remarkable that our patient achieved complete remission with the use of more accessible antifungal medications available in our region, such as caspofungin.

Despite guidelines of the Infectious Disease Society of America (https://www.idsociety.org/practice-guideline/practice-guidelines) that often recommend surgical intervention alongside antifungals for *Candida* endocarditis, our patient was successfully treated with caspofungin alone, suggesting that echinocandins may be effective alternatives even when surgery is not performed. Fungicidal echinocandins, like caspofungin, show strong activity against *Candida* biofilms [ 16 ]. The Infectious Disease Society of America recommends combined medical and surgical treatment for *Candida* endocarditis, typically using liposomal amphotericin B with or without flucytosine plus valve replacement, followed by six weeks of antifungal therapy [ 17 , 18 ]. However, caspofungin alone has achieved successful outcomes even without surgery, as reported in several cases  [ 16 , 19 - 21 ].

A limitation of this study is that the findings do not generalize treatment efficacy; rather, they highlight an important clinical scenario that warrants further investigation, especially concerning biofilm resistance in prosthetic valve infections.

## Conclusion

This case underscored the importance of combining molecular diagnostics with biofilm-specific antifungal susceptibility testing in managing complex *Candida* infections. The findings indicated that caspofungin is a potential alternative treatment for patients with *Candida* bloodstream infection and endocarditis who are not responsive to itraconazole. Further studies with larger sample sizes need to be conducted to conclude these findings comprehensively. 
